# Assessment of Wearable Device Adherence for Monitoring Physical Activity in Older Adults: Pilot Cohort Study

**DOI:** 10.2196/60209

**Published:** 2024-10-25

**Authors:** Huitong Ding, Kristi Ho, Edward Searls, Spencer Low, Zexu Li, Salman Rahman, Sanskruti Madan, Akwaugo Igwe, Zachary Popp, Alexa Burk, Huanmei Wu, Ying Ding, Phillip H Hwang, Ileana De Anda-Duran, Vijaya B Kolachalama, Katherine A Gifford, Ludy C Shih, Rhoda Au, Honghuang Lin

**Affiliations:** 1 Department of Anatomy and Neurobiology, Boston University Chobanian & Avedisian School of Medicine, Boston, MA, United States; 2 The Framingham Heart Study, Boston University Chobanian & Avedisian School of Medicine, Boston, MA, United States; 3 Department of Health Services Administration and Policy, Temple University College of Public Health, Philadelphia, PA, United States; 4 School of Information, The University of Texas at Austin, Austin, TX, United States; 5 Department of Epidemiology, Boston University School of Public Health, Boston, MA, United States; 6 School of Public Health and Tropical Medicine, Tulane University, New Orleans, LA, United States; 7 Department of Computer Science and Faculty of Computing & Data Sciences, Boston University, Boston, MA, United States; 8 Department of Medicine, Boston University Chobanian & Avedisian School of Medicine, Boston, MA, United States; 9 Vanderbilt Memory & Alzheimer’s Center, Department of Neurology, Vanderbilt University Medical Center, Nashville, TN, United States; 10 Beth Israel Deaconess Medical Center, Harvard Medical School, Boston, MA, United States; 11 Slone Epidemiology Center, Boston University Chobanian & Avedisian School of Medicine, Boston, MA, United States; 12 Departments of Neurology, Boston University Chobanian & Avedisian School of Medicine, Boston, MA, United States; 13 Department of Medicine, University of Massachusetts Chan Medical School, 55 Lake Avenue North, Worcester, MA, 01655, United States, 1 7744554881

**Keywords:** physical activity, remote monitoring, wearable device, adherence, older adults

## Abstract

**Background:**

Physical activity has emerged as a modifiable behavioral factor to improve cognitive function. However, research on adherence to remote monitoring of physical activity in older adults is limited.

**Objective:**

This study aimed to assess adherence to remote monitoring of physical activity in older adults within a pilot cohort from objective user data, providing insights for the scalability of such monitoring approaches in larger, more comprehensive future studies.

**Methods:**

This study included 22 participants from the Boston University Alzheimer’s Disease Research Center Clinical Core. These participants opted into wearing the Verisense watch as part of their everyday routine during 14-day intervals every 3 months. Eighteen continuous physical activity measures were assessed. Adherence was quantified daily and cumulatively across the follow-up period. The coefficient of variation was used as a key metric to assess data consistency across participants over multiple days. Day-to-day variability was estimated by calculating intraclass correlation coefficients using a 2-way random-effects model for the baseline, second, and third days.

**Results:**

Adherence to the study on a daily basis outperformed cumulative adherence levels. The median proportion of adherence days (wearing time surpassed 90% of the day) stood at 92.1%, with an IQR spanning from 86.9% to 98.4%. However, at the cumulative level, 32% (7/22) of participants in this study exhibited lower adherence, with the device worn on fewer than 4 days within the requested initial 14-day period. Five physical activity measures have high variability for some participants. Consistent activity data for 4 physical activity measures might be attainable with just a 3-day period of device use.

**Conclusions:**

This study revealed that while older adults generally showed high daily adherence to the wearable device, consistent usage across consecutive days proved difficult. These findings underline the effectiveness of wearables in monitoring physical activity in older populations and emphasize the ongoing necessity to simplify usage protocols and enhance user engagement to guarantee the collection of precise and comprehensive data.

## Introduction

The global demographic shift toward an aging population has become a pressing concern, with an increasing number of individuals being affected by cognitive disorders [1]. However, the limited number of effective treatments for cognitive diseases such as Alzheimer disease and other forms of dementia underscores the importance of early detection and monitoring of cognitive decline [2]. Early detection not only facilitates timely intervention but also allows for the management and possibly slowing of the progression of cognitive decline. Therefore, identifying modifiable risk factors plays a critical role in cognitive intervention strategies [3]. Among these, physical activity has emerged as a significant factor closely linked to health across many domains, including cardiovascular health [4] and cognitive functioning [5]. It has been recognized as one of the 12 modifiable risk factors for dementia [6]. A growing body of research indicates that regular physical activity can mitigate the risk of cognitive decline and improve brain function among older adults [7,8]. Moreover, meta-analyses have shown an association between increased physical activity and a reduced risk of dementia [9-11]. Such findings highlight the potential of evaluating and monitoring physical activity levels in older adults to improve detection and intervention of cognitive decline. By accurately capturing participants’ physical activity, actionable insights can be provided to inform lifestyle recommendations. This approach supports cognitive well-being and may help delay the onset of cognitive diseases.

Traditional in-clinic assessments of physical activity, however, are limited by their episodic nature, failing to consistently track an individual’s activity patterns over time. Additionally, these assessments are susceptible to modifications in gait when individuals are aware they are being observed, a phenomenon known as the Hawthorne effect [12,13]. In contrast, remote monitoring technologies offer a promising solution by enabling continuous and unobtrusive tracking of physical activity outside of clinical settings. This approach provides a more comprehensive picture of an individual’s physical activity patterns. This information is crucial for developing personalized interventions that are tailored specifically to the activity habits and needs of each participant. By identifying periods of inactivity or suboptimal activity levels, interventions can be more precisely targeted, thereby facilitating the effective implementation of strategies that are likely to be more engaging and beneficial for the individual. Therefore, our team has developed a pilot study using a participant-driven digital brain health platform [14], which incorporates wearable devices to collect physical activity data from participants. This initiative aims to facilitate comprehensive digital phenotyping of cognitive functions in the future.

However, monitoring physical activity continuously among older adults presents unique challenges, particularly concerning adherence issues [15]. Older adults may face physical, cognitive, or technological barriers that affect their consistent use of activity tracking devices [16]. These complexities require tailored approaches to ensure effective and sustained engagement in physical activity monitoring within this demographic. While some studies have focused on daily [17] and long-term [18] adherence to wearable devices, more studies that assess adherence metrics both daily and cumulatively throughout an entire study protocol could offer additional understanding of how older adults engage with these technologies. Additionally, this approach would be beneficial in exploring the consistency of the data collected, offering deeper insights into how consistently participants engage with the wearables over extended periods.

Therefore, this study aims to evaluate the adherence of wearable devices in remote monitoring of physical activity among older adults within a pilot study of digital brain health platform from the Boston University Alzheimer’s Disease Research Center (BU ADRC). Our objective is to evaluate older adults’ adherence at both daily and cumulative levels and the consistency of physical activity tracking and to explore preliminary strategies for optimizing study protocols, such as adjusting required wear durations. Our detailed examination of adherence patterns and device-specific data reliability aims to offer a unique addition to the collective understanding of wearable technology apps in aging populations.

## Methods

### Study Population

Participants from the Clinical Core of the BU ADRC were included in this study. The BU ADRC is one of around 33 ADRCs funded by the National Institute on Aging, sharing its findings with the National Alzheimer’s Coordinating Center to advance collaborative Alzheimer disease research. The BU ADRC is located in the urban area of Boston, focusing on the older adult population within this community. Detailed information about the ADRC has been documented in a prior study [19]. In 2021, the BU ADRC introduced a digital brain health platform incorporating various digital tools for data collection, such as wearable devices for monitoring physical activity [14]. The cognitive status of participants was determined through comprehensive consensus conferences involving multiple disciplines [20]. Additional information about these diagnostic procedures can be found in the previous study [21].

### Verisense Device

Verisense, developed by Shimmer Research Ltd, is a wrist-worn inertial measurement unit designed for tracking physical activity. This device integrates a tri-axial accelerometer and gyroscope, weighing 29.6 grams with dimensions of 35 mm × 43 mm × 12 mm, making it suitable for continuous wear on the wrist. With an IP55 resistance rating, the Verisense device is designed to be water-resistant and has protection against environmental contaminants and factors, safeguarding its functionality. Additionally, the device boasts a battery life of up to 6 months without the need for recharging, enhancing its usability for long-term monitoring. The sampling rate of the accelerometer of the Verisense device is 25 Hz. More description of Verisense can be found in a prior study [22]. Eighteen physical activity features (Table S1 in Multimedia Appendix 1), extracted using the GGIR software [23], were divided into 2 primary categories. The first category encompasses durations of various physical activities. These physical activities are classified by intensity levels from inactive to vigorous. The second category includes features related to acceleration, such as total acceleration during the most active 5 hours.

### Study Procedure

Participants were presented with available technologies from a digital brain health platform that spanned different test instrument options from smartphone apps to wearable devices with the level of use commitment for each defined [14]. Depending on participant preference, the presentation of technologies took place remotely over videoconference or in person. For in-person study visits, the Verisense device was configured and given to the participant during the visit. For remote visits, or quarterly check-ins following an in-person visit, the device was configured at the study site and then shipped to participants. Using a participant-centric study design, participants opted into the technologies of their choice. They were given a 2-week assessment period to use their selected technologies and assessment periods were scheduled at quarterly intervals. Participants who opted into Verisense were instructed to wear the tracker continuously over the 14-day period within a quarter. During the 14-day use period, there was no need to recharge the device, and thus could seamlessly integrate this monitoring tool into their routine activities for a comprehensive capture of physical activity data. Participants returned devices after the 14-day period. Every 3 months, the device was mailed back to them, and participants were given a reminder about using their technologies at the start of their 14-day assessment period, at the midpoint, and at the end. The physical activity data was retrieved from the Verisense cloud-based portal. The duration of this study was from September 2021 to February 2023. More information about the study procedures can be found in a previous publication [14].

### Statistical Analyses

Adherence metrics were derived from objective user data and analyzed daily, as well as cumulatively, for the first quarter of the 14-day follow-up period. First, daily adherence was evaluated by calculating the proportion of days where the daily wear rate surpassed predetermined thresholds relative to the total number of wear days. The thresholds denote the proportion of the days when participants wore the device. Given that participants maintain a high daily wear rate, we established thresholds of 90%, 95%, and 100% to differentiate levels of compliance throughout the day. Then, cumulative adherence was determined by assessing the overall proportion of days the device was worn (at least 8 hours a day [24]) during the requested 14-day period.

To assess the consistency of physical activity data across all participants over the entire study duration, we computed the within-person coefficient of variation (CV). This statistical parameter, defined as the ratio of the standard deviation to the mean of physical activity metrics, offers a uniform measure of data variability [25]. A lower CV value indicates greater consistency in the physical activity measure captured by the wearable devices.

We conducted a preliminary investigation to determine the potential for acquiring reliable physical activity data from participants within a shortened wear period. Specifically, we assessed the stability of physical activity measures through just 3 days of device use to increase the number of participants in the study samples. To measure the consistency of the data collected during these 3 consecutive wear days, including baseline, second, and third days between persons, the intraclass correlation coefficient (ICC) complemented by a 95% CI was calculated by a 2-way random-effects model [26].

### Ethical Considerations

The institutional review board of the Boston University Medical Campus approved the procedures and protocols of this study (H 405‐42). All participants provided written informed consent.

## Results

### Cohort Description

Our study included 22 participants from the BU ADRC (mean age 75, SD 7 years; 9/22, 41% women; an average of 16 years of education; Table 1). During the study period, 1 participant was diagnosed with non-amnestic, single-domain mild cognitive impairment. The distribution of each physical activity measure across 3 days, including 25th percentile, median, and 75th percentile values, is provided in Table S1 in Multimedia Appendix 1.

**Table 1.** Baseline demographics of the study participants (N=22).

Variable		Values
**Age (years), mean (SD)**		75 (7)
**Gender, n (%)**		
	Women	9 (41)
	Men	13 (59)
**Years of education, mean (SD)**		16 (2)
**Race, n (%)**		
	White	20 (91)
	Black or African American	2 (9)
**Current marital status, n (%)**		
	Married	16 (73)
	Divorced	5 (23)
	Never married	1 (4)
**Level of independence, n (%)**		
	Able to live independently	22 (100)
**Living situation, n (%)**		
	Lives alone	3 (14)
	Lives with spouse or partner	18 (82)
	Lives with relative or friend	1 (4)

### Study Adherence

Adherence to the study on a daily basis outperformed cumulative adherence levels. The median proportion of days where wear time surpassed 90% of total wear days stood at 92.1%, with an IQR spanning from 86.9% to 98.4%. As the threshold for daily wear duration increased, adherence notably declined. For days achieving 100% wear time, the median proportion dropped to 86.8%, with an IQR between 83.4% and 95.6% (Table 2).

Table 3 represents participant-level daily adherence to device usage at different compliance thresholds. It is evident that 5 participants maintain high adherence rates even as the thresholds increase, the overall daily adherence rates of 82% (18/22) participants tend to decrease as the threshold for daily wear time increases. At the 90% threshold, most of the participants show a high percentage of daily adherence, with many participants nearing or achieving full daily adherence. However, as the threshold increased to 95% and 100%, the number of days meeting the daily adherence criteria decreased. Table 4 represents participant-level cumulative adherence with the total number of worn days within the required initial 14-day period for each participant. There are varying levels of compliance across participants. Where 59% (13/22) of participants demonstrated high compliance, wearing the device for 10 or more days within the first quarter of the 14-day period, 32% (7/22) of participants exhibited notably lower compliance, with the device worn on fewer than 4 days. We also presented the daily adherence of participants to device usage during the initial 14-day period in Figure S1 in Multimedia Appendix 1.

**Table 2.** Summary of study adherence statistics at the population level.

Adherence level		Median (IQR)
**Daily**		
	Proportion of days with a daily wear rate above 90% of total wear days	92.1 (86.9-98.4)
	Proportion of days with a daily wear rate above 95% of total wear days	89.6 (83.4-95.6)
	Proportion of days with 100% daily wear rate of total wear days	86.8 (79.3-92.4)
**Cumulatively**		
	Proportion of wear days during the initial 14-day follow-up period	71.4 (21.4-78.6)

**Table 3.** Participant adherence to the wearable device usage at varying daily compliance thresholds.

Participant ID	Total wear days, n	Adherence days, n		
		Threshold: 90% daily wear rate	Threshold: 95% daily wear rate	Threshold: 100% daily wear rate
1	25	24	24	24
2	12	12	10	10
3	46	46	45	44
4	43	36	36	34
5	66	65	64	61
6	34	31	30	29
7	56	48	47	42
8	28	22	22	22
9	72	66	64	64
10	52	51	49	45
11	21	19	16	14
12	13	9	9	8
13	16	16	16	15
14	15	12	12	12
15	31	28	28	27
16	13	12	12	12
17	38	35	35	34
18	15	14	14	14
19	12	12	12	10
20	33	33	33	32
21	25	23	22	22
22	18	15	14	14

**Table 4.** Participant adherence to the device usage at the cumulative level.

Participant ID	Days worn within the initial 14-day period, n
1	8
2	11
3	11
4	11
5	10
6	1
7	10
8	3
9	11
10	3
11	5
12	1
13	1
14	12
15	10
16	1
17	10
18	12
19	12
20	11
21	12
22	3

### Data Consistency

**Figure 1.**
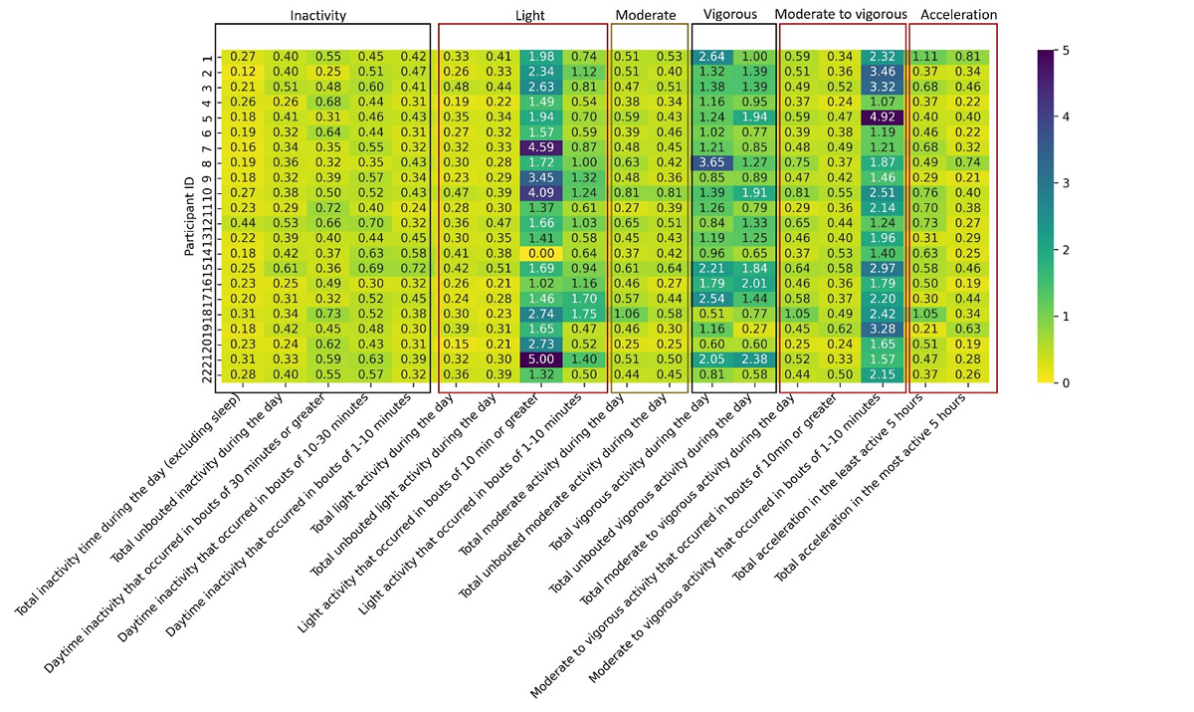
Variability of physical activity measures across participants. The vertical axis represents individual participants, with each row corresponding to 1 participant and their respective variability in physical activity measures. The coefficient of variation values are color coded, with darker shades indicating higher variability and lighter shades indicating lower variability.

Figure 1 displays the CV for different physical activity measures across participants. The vertical axis represents individual participants, with each row corresponding to 1 participant and their respective variability in physical activity measures. As shown by the heatmap, 4 physical activity measures—total vigorous activity during the day, light activity that occurred in bouts of 10 minutes or greater, moderate to vigorous activity that occurred in bouts of 1‐10 minutes, and moderate to vigorous activity that occurred in bouts of 10 minutes or greater—showed high variability (CV>1) for some participants, as evidenced by the darker shades in the respective columns. Conversely, other measures such as total inactivity time during the day displayed lower variability across participants, suggesting more consistency in those activity types.

Given that 32% (7/22) of participants wore the device for less than 4 days out of the initial required 14-day wear period, we conducted a preliminary investigation to determine if reliable data for specific physical activity measures could be obtained with just 3 days of wear. As shown in Table 5, four physical activity measures—total moderate activity during the day, total moderate to vigorous activity during the day, light activity that occurred in bouts of 1‐10 minutes, and daytime inactivity that occurred in bouts of 30 minutes or greater—demonstrated a moderate consistency with ICC greater than 0.5 over 3 wear days.

**Table 5.** Intraclass correlation coefficients of each physical activity measure for 2 different combinations of 3 wear days.

Physical activity measure		Baseline to day 2		Days 2 to 3	
		Values	*P* value	Values	*P* value
**Inactivity (minutes), ICC^a^ (95% CI)**					
	Total inactivity time during the day (excluding sleep)	0.44 (0.05 to 0.72)	.02	0.47 (0.09 to 0.74)	.008
	Total unbouted inactivity during the day	−0.07 (-0.49 to 0.37)	.62	0.32 (−0.08 to 0.64)	.06
	Daytime inactivity that occurred in bouts of 30 minutes or greater	*0.58^b^* (0.21 to 0.80)	.002	*0.53* (0.17 to 0.77)	.003
	Daytime inactivity that occurred in bouts of 10‐30 minutes	0.28 (−0.09 to 0.60)	.07	0.35 (−0.07 to 0.67)	.05
	Daytime inactivity that occurred in bouts of 1‐10 minutes	−0.17 (−0.55 to 0.27)	.77	0.06 (−0.37 to 0.46)	.40
**Light (minutes), ICC (95% CI)**					
	Total light activity during the day	0.29 (−0.15 to 0.63)	.10	0.68 (0.37 to 0.85)	<.001
	Total unbouted light activity during the day	0.29 (−0.15 to 0.64)	.09	0.54 (0.17 to 0.78)	.003
	Light activity that occurred in bouts of 10 minutes or greater	−0.03 (−0.43 to 0.39)	.55	0.61 (0.27 to 0.82)	<.001
	Light activity that occurred in bouts of 1‐10 minutes	*0.56* (0.18 to 0.79)	.003	*0.67* (0.37 to 0.85)	<.001
**Moderate (minutes), ICC (95% CI)**					
	Total moderate activity during the day	*0.55* (0.17 to 0.79)	.004	*0.61* (0.27 to 0.82)	<.001
	Total unbouted moderate activity during the day	0.49 (0.08 to 0.75)	.01	0.75 (0.48 to 0.89)	<.001
**Vigorous (minutes), ICC (95% CI)**					
	Total vigorous activity during the day	0.37 (−0.69 to 0.68)	.047	0.17 (−0.24 to 0.53)	.21
	Total unbouted vigorous activity during the day	0.84 (0.66 to 0.93)	<.001	−0.00 (−0.43 to 0.42)	.51
**Moderate to vigorous (minutes), ICC (95% CI)**					
	Total moderate to vigorous activity during the day	*0.55* (0.16 to 0.78)	.004	*0.60* (0.27 to 0.81)	<.001
	Moderate to vigorous activity that occurred in bouts of 10 minutes or greater	0.64 (0.30 to 0.83)	<.001	0.26 (−0.15 to 0.60)	.11
	Moderate to vigorous activity that occurred in bouts of 1‐10 minutes	0.51 (0.11 to 0.76)	.008	0.46 (0.08 to 0.73)	.009
**Acceleration, mg^c^ (95% CI)**					
	Total acceleration in the least active 5 hours	0.66 (0.34 to 0.84)	<.001	0.20 (−0.23 to 0.56)	.18
	Total acceleration in the most active 5 hours	0.49 (0.08 to 0.75)	.01	0.32 (−0.06 to 0.64)	.047

a ICC: intraclass correlation coefficient.

b Italic values represent ICC greater than 0.5 over 3 wear days.

c mg: milligee.

## Discussion

### Principal Findings

This study evaluated the adherence and consistency of remote monitoring physical activity among older adults. While adhering to a continuous 14-day device wear protocol posed challenges, the daily adherence levels were high, with many participants diligently following the usage guidelines. Furthermore, this study identified ICC values above 0.5 for certain physical activity measures, indicative of moderate reliability. While these results may not meet the highest standards for data stability, they demonstrate the feasibility of obtaining moderately reliable data with just 3 days of device wear. This insight supports the potential for shorter monitoring durations in future studies, which could reduce participant burden while still capturing consistent data.

Physical activity represents a modifiable behavioral factor with an association with enhanced outcomes across various health fields [27-29]. Yet, the deployment of physical activity interventions in clinical settings is not as prevalent as it could be [30]. Moreover, while participants may be encouraged or advised to increase their physical activity, healthcare professionals frequently face challenges in providing long-term follow-up [31]. This is particularly true for monitoring adherence to activity recommendations as outlined by the US Preventive Services Task Force [32]. Wearable technology presents an accessible approach to narrowing the divide between research and practical application in using physical activity as a preventive health strategy [33]. Especially, wrist-worn devices for monitoring physical activity are increasingly embraced by older adults [34]. The perspectives of older adults on activity trackers and their practical applications have been thoroughly documented, revealing generally high acceptability rates among this demographic [16]. However, whether older adults can strictly adhere to study protocols involving wearable devices still requires further investigation. In this study, participants are required to return the device after completing a 14-day wear period. This process assists in recalibrating the device and checking its functionality. Additionally, research indicated that the usage rates of wrist-worn devices decline over time after they are distributed to participants [35]. Therefore, returning the device acts as a reminder for participants, encouraging continued adherence to the usage protocol. While other reminder methods are available, this study opts for device return as the chosen strategy.

This study examined the adherence at both daily and cumulative levels. Overall, study adherence daily outperformed cumulative adherence levels. The median proportion of days where wear time surpassed 90% of total wear days stood at 92.1%, indicating that a substantial proportion of participants comply well with the device wear requirements at the daily level. However, ensuring near-perfect or perfect daily adherence remains a challenge, as seen in the decrease in adherence days at the strictest threshold of 100%. However, at the cumulative level, 32% (7/22) of participants in this study exhibited notably lower adherence, with the device worn on fewer than 4 days within the requested initial 14-day period. While 5 participants adhered to the protocol of recording their activity every 3 months, discrepancies were observed, with 5 participants having intervals of device use exceeding the 3-month interval. This variability signals that while some participants readily integrate the wearable devices into their daily routines, others face barriers that hinder consistent use. These barriers emphasize the necessity for adaptable and personalized approaches in encouraging sustained device usage. We also explored the implication of marital status, level of independence, and living situation on the study adherence. Three participants lived alone, two of which demonstrated relatively good adherence. We did not observe distinct patterns in adherence among participants with different marital statuses. Acknowledging and addressing these individual needs are pivotal in ensuring the efficacy of wearable technology as a tool for health monitoring in research settings.

Given that 32% (7/22) of participants wore the device for less than 4 days out of the requested 14-day wear period, we conducted a preliminary investigation to determine if some physical activity measures with moderate reliability (ICC bigger than 0.5) [36] could be obtained with just 3 days of wear. This investigation revealed that specific physical activity measures demonstrating moderate reliability, with ICC values greater than 0.5, can be reliably captured within this shorter timeframe. However, the overall reliability of these measures is not as strong as desired, suggesting that extended wear durations may still be necessary to ensure comprehensive data reliability across a broader range of physical activities. While this exploratory study suggests that shorter wear durations might reduce participant burden and enhance data collection efficiency for specific activities, it also highlights the need for careful result interpretation. Future research should prioritize enhancing the reliability of these measures before considering reduced monitoring durations to ensure that the data collected remains both robust and reliable. Our study employed the Verisense watch, a wearable technology that captures a comprehensive array of physical activity measures, offering a valuable data resource for further research into cognitive impairment. This pilot study not only assesses the adherence of older adults to a long-lasting, waterproof wearable device but also evaluates the consistency of detailed physical activity measures. As part of our ongoing digital brain health platform, we aim to integrate these physical activity insights with other modalities such as sleep and digital cognitive assessments to enhance our understanding of cognitive health. With an anticipated increase in sample size, we plan to provide a more complete digital phenotyping of cognition that leverages the combined strengths of various data types.

We recognized a few limitations in our study. First, the relatively small sample size makes this study serving primarily to offer preliminary insights at a pilot study level. Second, there is a potential for selection bias to influence the findings of this study. Specifically, within the digital brain health platform, participants are given the freedom to choose from various technologies according to their comfort and commitment levels. This choice means that participants who are more comfortable using the Verisense device might be more likely to participate in this study. Further research is also necessary to investigate the reasons behind the instances when the devices were taken off by participants. Third, investigating the impact of different wear time thresholds for defining worn days could be a valuable direction for future research, particularly in assessing device compliance and changes in physical activity measures. Fourth, the consistent inactivity observed in certain participants underscores the necessity for in-depth investigations into the potential health implications or obstacles preventing adherence, as well as the causes of activity variability. Future research should collect more comprehensive lifestyle and social determinant data to explain it.

### Conclusion

In summary, this study revealed that while older adults generally showed high daily adherence to the wearable device, consistent usage across consecutive days proved difficult. The varied adherence rates highlight the importance of tailored strategies to improve commitment to the study. Additionally, our initial analysis suggests that stable data for specific activities can be achieved with as little as 3 days of device wear, opening the door to potentially shorter required wear times in subsequent studies. These findings underline the effectiveness of wearables in monitoring physical activity in older populations and emphasize the ongoing necessity to refine usage protocols and enhance user engagement to guarantee the collection of precise and comprehensive data.

## Supplementary material

10.2196/60209 Multimedia Appendix 1 Supplementary table and figure.

## Abbreviations

BU ADRC: Boston University Alzheimer’s Disease Research Center
CV: coefficient of variation
ICC: intraclass correlation coefficient

## References

[1] Pais R, Ruano L, Carvalho OP, Barros H (2020). Global cognitive impairment prevalence and incidence in community dwelling older adults—a systematic review. Geriatrics (Basel).

[2] Zucchella C, Sinforiani E, Tamburin S (2018). The multidisciplinary approach to Alzheimer's disease and dementia: a narrative review of non-pharmacological treatment. Front Neurol.

[3] Zhang XX, Tian Y, Wang ZT, Ma YH, Tan L, Yu JT (2021). The epidemiology of Alzheimer’s disease modifiable risk factors and prevention. J Prev Alzheimers Dis.

[4] Warburton DER, Bredin SSD (2017). Health benefits of physical activity: a systematic review of current systematic reviews. Curr Opin Cardiol.

[5] Dominguez LJ, Veronese N, Vernuccio L (2021). Nutrition, physical activity, and other lifestyle factors in the prevention of cognitive decline and dementia. Nutrients.

[6] Livingston G, Huntley J, Sommerlad A (2020). Dementia prevention, intervention, and care: 2020 report of the Lancet Commission. Lancet.

[7] Biazus-Sehn LF, Schuch FB, Firth J, de Souza Stigger F (2020). Effects of physical exercise on cognitive function of older adults with mild cognitive impairment: a systematic review and meta-analysis. Arch Gerontol Geriatr.

[8] Law CK, Lam FM, Chung RC, Pang MY (2020). Physical exercise attenuates cognitive decline and reduces behavioural problems in people with mild cognitive impairment and dementia: a systematic review. J Physiother.

[9] Hamer M, Chida Y (2009). Physical activity and risk of neurodegenerative disease: a systematic review of prospective evidence. Psychol Med.

[10] Blondell SJ, Hammersley-Mather R, Veerman JL (2014). Does physical activity prevent cognitive decline and dementia? A systematic review and meta-analysis of longitudinal studies. BMC Public Health.

[11] Guure CB, Ibrahim NA, Adam MB, Said SM (2017). Impact of physical activity on cognitive decline, dementia, and its subtypes: meta-analysis of prospective studies. Biomed Res Int.

[12] McCambridge J, Witton J, Elbourne DR (2014). Systematic review of the Hawthorne effect: new concepts are needed to study research participation effects. J Clin Epidemiol.

[13] Czech MD, Psaltos D, Zhang H (2020). Age and environment-related differences in gait in healthy adults using wearables. NPJ Digit Med.

[14] De Anda-Duran I, Hwang PH, Popp ZT (2023). Matching science to reality: how to deploy a participant-driven digital brain health platform. Front Dement.

[15] Valenzuela T, Okubo Y, Woodbury A, Lord SR, Delbaere K (2018). Adherence to technology-based exercise programs in older adults: a systematic review. J Geriatr Phys Ther.

[16] Moore K, O’Shea E, Kenny L (2021). Older adults’ experiences with using wearable devices: qualitative systematic review and meta-synthesis. JMIR Mhealth Uhealth.

[17] Paolillo EW, Lee SY, VandeBunte A (2022). Wearable use in an observational study among older adults: adherence, feasibility, and effects of clinicodemographic factors. Front Digit Health.

[18] Yingling LR, Mitchell V, Ayers CR (2017). Adherence with physical activity monitoring wearable devices in a community-based population: observations from the Washington, D.C., Cardiovascular Health and Needs Assessment. Transl Behav Med.

[19] Frank B, Ally M, Brekke B (2022). Plasma p-tau_181_ shows stronger network association to Alzheimer’s disease dementia than neurofilament light and total tau. Alz Dement.

[20] Steenland K, Macneil J, Bartell S, Lah J (2010). Analyses of diagnostic patterns at 30 Alzheimer’s disease centers in the US. Neuroepidemiology.

[21] Leoutsakos JMS, Forrester SN, Lyketsos C, Smith GS (2015). Latent classes of neuropsychiatric symptoms in NACC controls and conversion to mild cognitive impairment or dementia. J Alzheimers Dis.

[22] McDevitt B, Moore L, Akhtar N, Connolly J, Doherty R, Scott W (2021). Validity of a novel research-grade physical activity and sleep monitor for continuous remote patient monitoring. Sensors (Basel).

[23] Migueles JH, Rowlands AV, Huber F, Sabia S, van Hees VT (2019). GGIR: a research community–driven open source R package for generating physical activity and sleep outcomes from multi-day raw accelerometer data. J Meas Phys Behav.

[24] Evenson KR, Wen F, Metzger JS, Herring AH (2015). Physical activity and sedentary behavior patterns using accelerometry from a national sample of United States adults. Int J Behav Nutr Phys Act.

[25] Shechtman O (2013). Methods of Clinical Epidemiology.

[26] Liljequist D, Elfving B, Roaldsen KS (2019). Intraclass correlation: a discussion and demonstration of basic features. PLoS One.

[27] Bauman AE, Sallis JF, Dzewaltowski DA, Owen N (2002). Toward a better understanding of the influences on physical activity: the role of determinants, correlates, causal variables, mediators, moderators, and confounders. Am J Prev Med.

[28] Chekroud SR, Gueorguieva R, Zheutlin AB (2018). Association between physical exercise and mental health in 1.2 million individuals in the USA between 2011 and 2015: a cross-sectional study. Lancet Psychiatry.

[29] Bouchard C, Blair SN, Haskell WL (2012). Physical Activity and Health.

[30] Koorts H, Eakin E, Estabrooks P, Timperio A, Salmon J, Bauman A (2018). Implementation and scale up of population physical activity interventions for clinical and community settings: the PRACTIS guide. Int J Behav Nutr Phys Act.

[31] Middleton KR, Anton SD, Perri MG (2013). Long-term adherence to health behavior change. Am J Lifestyle Med.

[32] Bleich SN (2022). Updated USPSTF recommendations for behavioral counseling interventions: gaps, challenges, and opportunities. JAMA Intern Med.

[33] Böhm B, Karwiese SD, Böhm H, Oberhoffer R (2019). Effects of mobile health including wearable activity trackers to increase physical activity outcomes among healthy children and adolescents: systematic review. JMIR Mhealth Uhealth.

[34] Teixeira E, Fonseca H, Diniz-Sousa F (2021). Wearable devices for physical activity and healthcare monitoring in elderly people: a critical review. Geriatrics (Basel).

[35] McManus DD, Trinquart L, Benjamin EJ (2019). Design and preliminary findings from a new electronic cohort embedded in the Framingham Heart Study. J Med Internet Res.

[36] Koo TK, Li MY (2016). A guideline of selecting and reporting intraclass correlation coefficients for reliability research. J Chiropr Med.

