# Supplementary material for: Assessment of Wearable Device Adherence for Monitoring Physical Activity in Older Adults: Pilot Cohort Study
Source: JMIR Aging. 2024 Oct 25;7:e60209. doi: 10.2196/60209 (PMC11530080; doi:10.2196/60209)

**Table S1**. The distribution of physical activity measures in three consecutive wear days.

|  | **Physical activity measure** | | |  |  | **Baseline** | **2^nd^ day** | **3^rd^ day** |
| --- | --- | --- | --- | --- | --- | --- | --- | --- |
|  | Inactivity | Total inactivity time during day (excluding sleep), minute |  |  | 522.17 [406.9, 735.75] | | 582.67 [491.30, 647.20] | 634.04 [571.80, 729.3] |
|  |  | Total unbouted inactivity during the day, minute |  |  | 85.92 [53.02, 106.79] | | 75.67 [49.48, 121.90] | 97.92 [73.08, 133.06] |
|  |  | Daytime inactivity that occurred in bouts of 30 minutes or greater, minute |  |  | 232.08 [158.50, 477.70] | | 310.04 [181.30, 413.20] | 312.08 [243.90, 486.80] |
|  |  | Daytime inactivity that occurred in bouts of 10-30 minutes, minute |  |  | 67.54 [14.44, 93.73] | | 97.63 [68.29, 127.46] | 88.13 [46.04, 111.48] |
|  |  | Daytime inactivity that occurred in bouts of 1-10 minutes, minute |  |  | 94.71 [56.90, 120.08] | | 89.29 [69.00, 128.75] | 106.33 [79.79, 132.1] |
|  | Light | Total light activity during the day, minute |  |  | 257.71 [178.31, 308.65] | | 224.42 [156.02, 318.48] | 215.33 [172.10, 307.5] |
|  |  | Total unbouted light activity during the day, minute |  |  | 109.00 [65.50, 147.4] | | 105.38 [62.08, 138.88] | 121.96 [84.4, 143.6] |
|  |  | Light activity that occurred in bouts of 10 min or greater, minute |  |  | 18.08 [0, 60.13] | | 10.17[0, 38.35] | 5.04 [0, 27.94] |
|  |  | Light activity that occurred in bouts of 1-10 minutes, minute |  |  | 74.83 [39.17, 107.92] | | 65.79 [44.06, 104.00] | 61.38 [48.12, 123.17] |
|  | Moderate | Total moderate activity during the day, minute |  |  | 61.33 [27.71, 95.60] | | 45.29 [34.71, 106.77] | 45.46 [31.98, 65.71] |
|  |  | Total unbouted moderate activity during the day, minute |  |  | 28.83 [15.44, 56.69] | | 28.83 [14.79, 60.67] | 32.58 [18.88, 46.21] |
|  | Vigorous | Total vigorous activity during the day, minute |  |  | 0.58 [0.10, 1.44] | | 0.63 [0.19, 1.79] | 0.42 [0.19, 090] |
|  |  | Total unbouted vigorous activity during the day, minute |  |  | 0.29 [0.08, 0.79] | | 0.25 [0.08, 0.42] | 0.21 [0.08, 0.60] |
|  | Moderate to vigorou | Total moderate to vigorous activity during the day, minute |  |  | 61.46 [28.35, 102.13] | | 53.00 [34.79, 108.04] | 46.50 [31.98, 66.31] |
|  |  | Moderate to vigorous activity that occurred in bouts of 10min or greater, minute |  |  | 0 [0, 11.38] | | 0 0, [25.35] | 0 [0, 7.5] |
|  |  | Moderate to vigorous activity that occurred in bouts of 1-10 minutes, minute |  |  | 8.625 [2.33, 18.38] | | 12.25 [4.63, 24.42] | 7.0835 [3.60, 13.92] |
|  | Acceleration | Total acceleration in the least active 5 hours, mg |  |  | 5.65 [3.44, 7.18] | | 5.92 [2.78, 8.97] | 4.56 [3.06, 6.79] |
|  |  | Total acceleration in the most active 5 hours, mg |  |  | 48.88 [33.49, 64.90] | | 50.49 [34.89, 63.11] | 38.93 [31.47, 48.50] |

 Note: The median (IQR) values were presented for each physical activity measures. Milligee: mg.

**Figure S1**. Participant adherence to the device usage at daily level with different compliance thresholds during the initial 14-day period. The series of bar charts represent the adherence of participants to the device usage at varying thresholds, including 90%, 95%, and 100%. Each bar chart corresponds to a different threshold level, displaying a side-by-side comparison of the total number of days each participant wore the device during the initial 14-day period (blue bars) against the number of days they met the specified adherence criteria (orange bars).


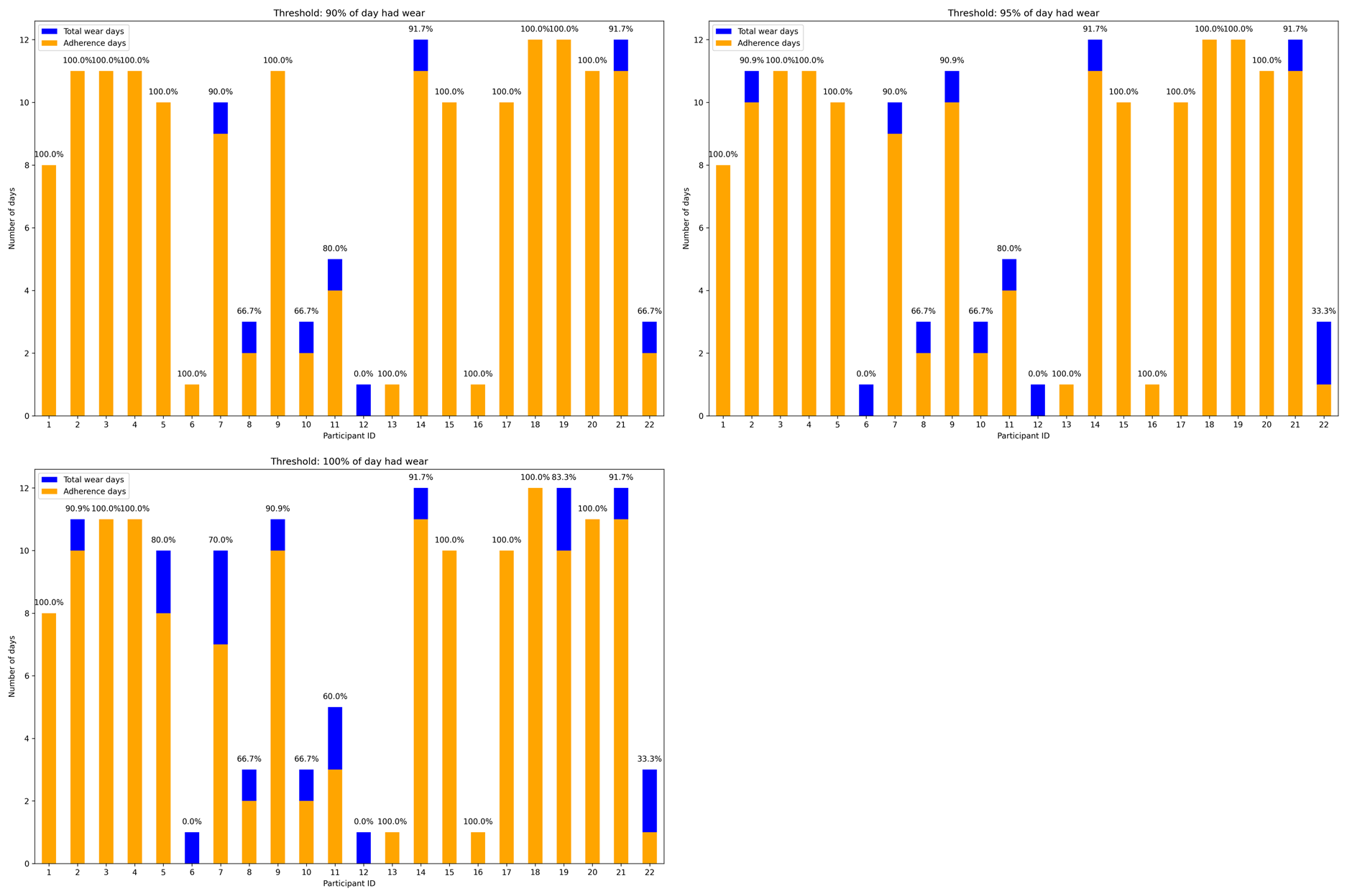

Supplement: Multimedia Appendix 1 [file aging-v7-e60209-s001.docx]
